# Factor graph-aggregated heterogeneous network embedding for disease-gene association prediction

**DOI:** 10.1186/s12859-021-04099-3

**Published:** 2021-03-29

**Authors:** Ming He, Chen Huang, Bo Liu, Yadong Wang, Junyi Li

**Affiliations:** 1 School of Computer Science and Technology, Harbin Institute of Technology (Shenzhen), Shenzhen, 518055 Guangdong China; 2 Center for Bioinformatics, School of Computer Science and Technology, Harbin Institute of Technology, Harbin, 150001 Heilongjiang China

**Keywords:** Disease-gene association prediction, Heterogeneous network, Graph neural network, Factorization

## Abstract

**Background:**

Exploring the relationship between disease and gene is of great significance for understanding the pathogenesis of disease and developing corresponding therapeutic measures. The prediction of disease-gene association by computational methods accelerates the process.

**Results:**

Many existing methods cannot fully utilize the multi-dimensional biological entity relationship to predict disease-gene association due to multi-source heterogeneous data. This paper proposes FactorHNE, a factor graph-aggregated heterogeneous network embedding method for disease-gene association prediction, which captures a variety of semantic relationships between the heterogeneous nodes by factorization. It produces different semantic factor graphs and effectively aggregates a variety of semantic relationships, by using end-to-end multi-perspectives loss function to optimize model. Then it produces good nodes embedding to prediction disease-gene association.

**Conclusions:**

Experimental verification and analysis show FactorHNE has better performance and scalability than the existing models. It also has good interpretability and can be extended to large-scale biomedical network data analysis.

## Background

In the field of biomedical research, the disease-gene association prediction is a fundamental and important problem [1, 2]. With the advancement of machine learning and artificial intelligence research, many machine learning methods have been applied to discover new genetic associations of diseases. However, there are still many challenges in this research area. For instance, the number of gene sets is much larger than that of confirmed disease-related genes. In other words, it is difficult to use less data to mine the pattern of disease-gene association. Meanwhile, the genetic heterogeneity of diseases makes the pattern diverse, which increases the difficulty of mining too. Then it is suggested that if a gene has similar characteristics to a known disease causal gene, it might also be associated with this same disease.

Disease-gene association prediction is a process of mining and discovering candidate genes that may be associated with disease through the data set of known actual disease-gene association. And computational methods can greatly accelerate the process of research in the field of biological information. In recent years, a lot of related work emerge. Some works [3, 4] are from data sources (such as gene expression data, KEGG, etc.) to manually extract features, then uses machine learning classifier to train and predict the task. However, the amount of data is very large, and not all genes have the same extent of exploration. Therefore, except for some common genes, most of the available data are very scarce, which makes feature engineering only use a small number of common features. Biological data is complex, and various biological data have become a simple and clear form through network representation, so network-based methods [5] have become the mainstream direction of disease-gene association prediction. These methods [6–8] are used to mine new disease-gene association in biological entity network, and have achieved good performance. However, due to the limited number of data sources, each method can be further improved through an integrated process. In particular, Han et al. [9] predicted the new disease-gene association through the graph convolution network with the features obtained by the integrated matrix decomposition method and the original features, which can capture the linear and nonlinear relationship between diseases and genes at the same time and obtain better performance. Yang et al. [8] integrated from the data level, carefully selected the most favorable data sources to build a multi-mode network, and used Node2vec [10] to learn node representation on complex network, so as to measure the proximity of disease-gene node pairs and make prediction, or reconstruct a two-layer heterogeneous network containing only disease and gene nodes, which can be used for the final network prediction methods. Benefit from the rapid development of graph neural network, the ability of learning node representation for downstream tasks (link prediction, etc.) directly from heterogeneous networks is greatly enhanced. At present, data level integration is adopted in many works to avoid excessive information loss in the process of model integration (a single model cannot capture all the feature information).

Many methods are only applicable to scenes of homogeneous networks. In fact, most of the scenes in real life are modeled as heterogeneous networks, that is, including multiple node types and multiple relationship types. For example, DeepWalk [11], Node2vec [10], LINE [12] and other methods are designed for the purpose of passing through the network. If they are applied to heterogeneous network data, the heterogeneity of nodes and relationships will be ignored, thus rich semantic information will be lost. Therefore, it is urgent to develop heterogeneous network representation learning methods. Metapath2vec [13] is one of the first methods of representation learning from heterogeneous networks, and also the first propose place of metapath, where rich semantic information between different nodes is contain, but it is similar to those in the case of Node2vec, where it is not extendable, and relies on the structural integrity of the network. Wang et al. [14] proposed HAN, based on the GAT [15] model of integrating a neighbor's graph neural network using a self-attention mechanism, as well as metapath, based on the multiple sampling of neighbors, using a self-attention mechanism to integrate neighbor information from metapath, and after the addition of a layer of semantic attention, which can fusion multiple node information from the different metapath pattern. Since it can alleviate the problem of network heterogeneity and generate node representation containing rich topological structure information and semantic information, this architecture has become a classic architecture of graph neural network model for heterogeneous network. Subsequently, various heterogeneous network embedding models basically retain the two-layer attention mechanism of HAN. However, most of the existing models consider only two end node and one edge from metapath, ignoring information from multiple nodes in the metapath intermedia, which lead to a problem known as early summarization [16].

In this work, we present FactorHNE, which is a heterogeneous graph neural network model architecture for aggregating multiple factor graphs for prediction tasks. In addition to any information available, a heterogeneous network of four different nodes was constructed, and based on metapath, multiple patterns were mined. In the node information aggregation phase, in order to alleviate the problem of early summarization, we used factor diagram decomposition based on metapath reconstruction of neighborhood subgraphs to capture the multiple semantics included in the metapath relationship, and the effectiveness of this method was verified [17], when multiple relationships between nodes in the graph were mined. After any of the node features are mine in metapath, we used an attention mechanism to integrate the semantic information in any of the metapath. By designing this model, we can make good use of the multi-source biological data to mine the pattern of disease-gene association and promote the understanding of disease pathogenesis and the development of therapeutic drugs. Our major contributions can be summarized as follows:

- Through factor decomposition of neighborhood subgraphs of nodes, we mined a variety of relationship information, and effectively alleviated the early summarization problem from metapath sampling.
- We designed a number of comparison experiments on a large-scale network, verified the performance advantage of our model over existing models, and analyzed the experimental results.
- We designed a deep learning model for heterogeneous network link prediction, which can effectively learn rich topological information and semantic information in heterogeneous networks, and can be extended to large-scale biomedical network data, and verified by design experiments.

## Results and discussions

In this section, we will introduce our experimental settings and result analysis in detail. At the same time, FactorHNE and other network embedding methods are compared under fair conditions. By observing and comparing the experimental results, the advantages of our model in the task of disease-gene association prediction in a large-scale heterogeneous network are analyzed.

## Baselines

To assess the performance of a link prediction model, we adopt the AP, AUC, Precision@K, and Recall@K which commonly used in model evaluation. AP represents the area under the P–R curve drawn according to the precision and recall of the model, AUC represents the area under ROC curve of the model, these two indicators are commonly used to evaluate prediction tasks, in addition, the Precision@K and Recall@K denote the precision and recall are producted based on the Kth largest threshold. We calculate these evaluation indexes for FactorHNE model and other baselines, and then analyze the experimental results. And the baseline we use are shown as follows:

- Metapath2vec [13] is a traditional random walk based model, using metapath to mentor the next hop neighbor, producing a heterogeneous node sequence based on the specific metapath, and using the Skip-gram model to generate node embedding, we have tried a variety of metapath, report the best of the results.
- HIN2vec [18] is a model for heterogeneous network embedding. By applying optimization constraints to multiple downstream tasks between node pairs, it is possible to train both heterogeneous nodes embedding and multiple metapath embedding, meaning that it will automatically try any combination of metapath to produce the most suitable node embedding.
- HERec [19] is a recommended model for heterogeneous networks, based on multiple metapath pattern converts the original heterogeneous network to an homogeneous network, then use DeepWalk model to generate node embedding from all metapath, and after combined ones from each metapath, a final embedding of the node will be generated.
- GAT [15] is a GNN model for homogeneous networks, where neighbor information is aggregated using a self-attention mechanism, and node embedding is obtained using semi-supervise training, it is an end-to-end model. where we show the best after attempted multiple metapath pattern.
- HAN [14] is a GNN model for heterogeneous networks, using methods similar to those found in HERec to convert networks to homogeneous, then using GAT to generate node embedding in each metapath semantic environment, and finally using a semantic-level attention to aggregate node embedding in different metapath pattern.
- MAGNN [20] is a heterogeneous network GNN model, which alleviates the early summarization problem to some extent by encoding metapath instances, and extends the model to larger heterogeneous networks through neighbor sampling mechanism.

All baselines can be further subdivided into unsupervised and semi supervised learning models. The first three models belong to unsupervised learning model, and the last three GNN models belong to semi supervised learning model. However, our dataset does not have node label information, so we add a loss function based on downstream link prediction task to GNN model, For the traditional model (the first three baselines), we set the parameters of random walk as follows: window set is equal to 5, walk length is equal to 100, each node performs 10 walks, and the number of negative samples is 5, We set the embedding dimension of the generation node to 64. We use the Adam optimizer with a learning rate of 0.005 and a L2 penalty weight of 0.001. We use the same training set and test set, and the same training method. For GNN models (MAGNN and FactorHNE) using neighbor sampling, the number of neighbors is fixed to 100. For fair comparison, our results take the average value of three runs.

## Experiment analysis

The experimental results of FactorHNE and other baselines are shown in Table 1. Through the analysis of the experimental results, some conclusions can be drawn.

**Table 1:** Experimental results (%) of link prediction task on dataset

Model	P@1000	R@1000	P@10,000	R@10,000	P@20,000	R@20,000
Metapath2vec	99.60 ± 0.202	3.81 ± 0.301	95.40 ± 0.202	36.46 ± 0.023	82.65 ± 0.011	63.18 ± 0.022
HIN2vec	99.60 ± 0.051	3.81 ± 0.241	74.22 ± 0.102	28.37 ± 0.043	58.99 ± 0.031	45.09 ± 0.021
HERec	63.30 ± 0.063	2.42 ± 0.182	72.58 ± 0.035	27.74 ± 0.061	71.55 ± 0.102	54.69 ± 0.100
GAT	94.90 ± 0.171	3.62 ± 0.304	93.67 ± 0.043	35.80 ± 0.102	90.45 ± 0.211	69.14 ± 0.117
HAN	99.60 ± 0.093	3.81 ± 0.301	**99.16** ± 0.202	**37.90** ± 0.306	96.28 ± 0.130	73.59 ± 0.317
MAGNN	99.30 ± 0.122	3.80 ± 0.103	98.11 ± 0.351	37.50 ± 0.203	94.99 ± 0.033	72.61 ± 0.091
FactorHNE	**99.70** ± 0.062	**3.81** ± 0.202	99.07 ± 0.121	37.87 ± 0.082	**96.91** ± 0.072	**74.08** ± 0.145

Bold values are the highest value of all baselines

**Fig. 1:**
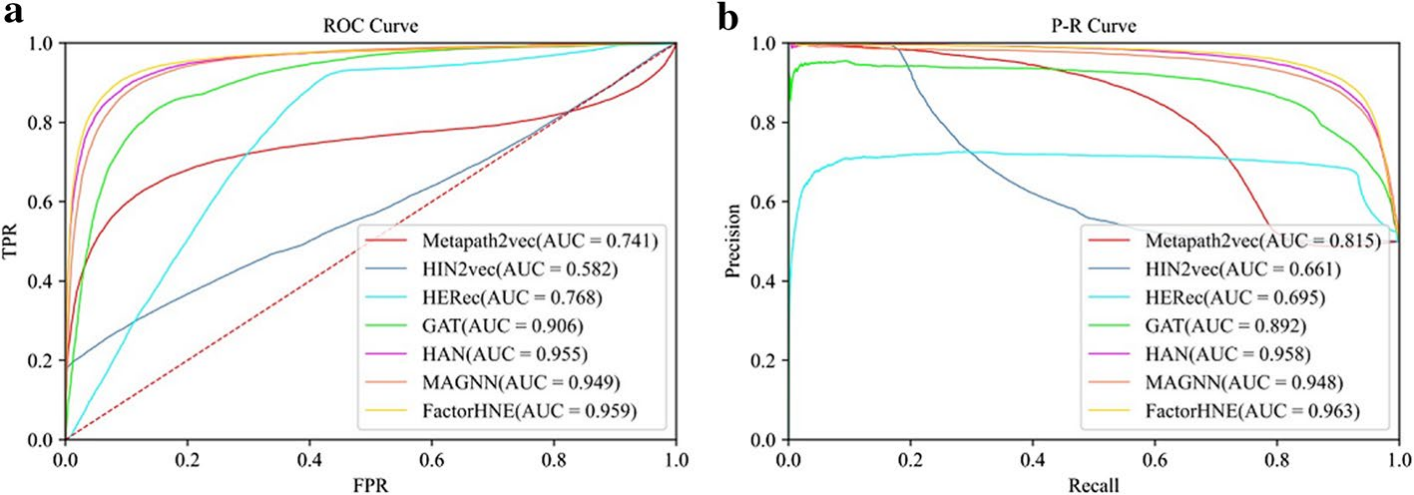
**a** ROC curves of all models; **b** P–R curve of all models

ROC and PR curves are shown in Fig. 1. Our test set contains 52,328 positive and negative edge samples(generate negative samples by randomly selecting edges that do not exist in the dataset), so the value of K in Precision@K and Recall@K is {1000, 10,000, 20,000}. As can be seen from Table 1, the performance of the traditional model based on unsupervised learning is much lower than that of the GNN model based on semi supervised learning. The main reason is that they cannot carry out end-to-end learning and cannot benefit from the gradient optimization of downstream tasks. Therefore, the embedding generated may be stable in most of the downstream tasks, but none of them will be particularly excellent. Another reason is that they can only use the topology information in the network, ignoring the content of the node itself. Therefore, compared with the GNN model, the performance gap will be more obvious. After all, the information contained is not in the same level.

In addition, by comparing the performance of several GNN models, it can be found that the GAT model designed for homogeneous network has great advantages over the traditional model, but compared with FactorHNE and HAN, it is still about 5–6% worse in performance. In addition, it seems that the improved metapath instance encode component of MAGNN based on HAN does not seem to be particularly obvious in our problem, because MAGNN uses the strategy of sampling neighbors to improve scalability like FactorHNE, and the actual number of neighbors may exceed several orders of magnitude. The reason for poor performance may be that the improvement brought by the improvement is not enough to make up for the loss of neighbor information Missing gap. In contrast, our FactorHNE model uses the strategy of aggregation factor graph to mine multiple semantic information implied in metapath. At the same time, it uses sampling neighbor strategy to improve the scalability of the model, and even outperforms HAN which uses more neighbor information. In a word, compared with the improved strategy of MAGNN, our strategy of aggregation factor graph shows a good effect in solving early summation issue, and its performance is also ahead of all baselines in most indicators.

### Experiment analysis

**Fig. 2:**
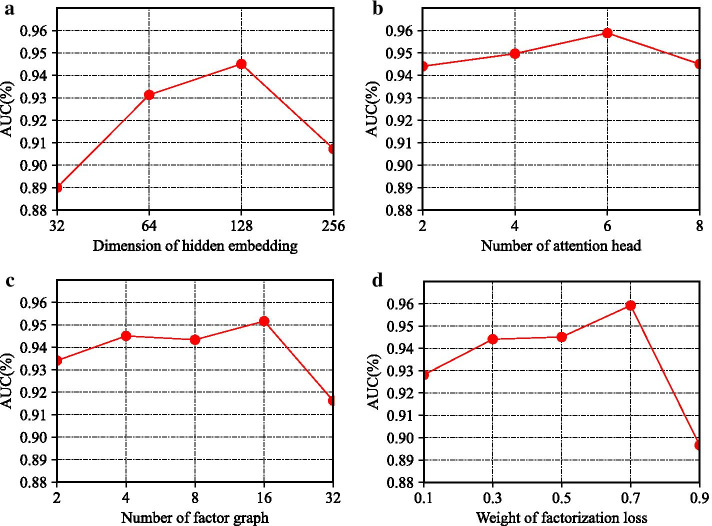
Parameter analysis of FactorHNE

In this section, we will fine tune the values of the four parameters, compare the performance changes of the model under different parameters, and measure the change degree of the model through the AUC index. The comparative experimental results of all parameters are shown in Fig. 5. The four parameters are as follows:

- *Dimension of hidden embedding* Figure 2a shows the influence of dimension of hidden embedding on the final performance of the model. We can see that the curve rises rapidly at the beginning, achieves the best result when reaching 128, and then begins to decline. We think that this is because the aggregation of multiple factor graphs requires a larger dimension to contain rich information, while the latter descending part may contain redundant dimensions, which produces noise.
- *Number of attention head* In Fig. 2b, we verify the influence degree of the long attention mechanism. We can see that the curve is relatively gentle, and there is a slow upward trend at the beginning. Therefore, the long attention mechanism has a certain improvement effect on the model, and ensures that the model is more stable, which is conducive to the recurrence of the results.
- *Number of factor graph* This parameter represents the number of factor graphs we use when factoring neighborhood subgraphs. In Fig. 2c, the curve is basically smooth, and the best performance is achieved at 16. Considering the computational overhead caused by increasing the number of factor graphs, the best performance can be achieved by using fewer factor graphs, and it does not need to adjust parameters to get better performance.
- *Weight of factorization loss* This parameter is used to control the proportion of downstream task loss and factor graph decomposition loss. From Fig. 2d, we find that the AUC can be improved by 2–3% by increasing the decomposition loss of factor graph within a certain range, which indicates that the multiple semantic information mined by factor graph has a beneficial effect on the prediction performance of the model. However, excessive increase in the weight of γ will make a mockery of the impact of downstream task loss, resulting in performance degradation.

### Case study

In order to further analyze the biological significance of our model, we select two different diseases for mining analysis. The two different disease are Gait abnormality (CUI:C0575081) and Congenital Epicanthus (CUI:C0678230). We use our model with optimal performance to calculate the score of possible association between these two diseases and all genes in the dataset, and listed the top 20 candidate genes. The results are shown in Table 2.

**Table 2:** Case study results

CUI	Disease name	CUI	Disease
C0575081	Gait abnormality	CUI:C0678230	Congenital Epicanthus

Gene	Original DB	Gene	Original DB
FGFR3		FBN1	
POU1F1		IKBKG	
ROR2		LBR	
GRM1		KCNJ2	
OFD1	DisGeNet*	ROR2	DisGeNet
BMPR1B		SOX9	
FGFR2		FLNA	DisGeNet*
RPS29		GDF5	
IL6		LMNA	
SLC9A6		TBX3	
MYH6		FOXG1	DisGeNet
RAF1		OFD1	DisGeNet
COL6A2		HDAC6	
MKKS		GRM1	
MAP3K7		TGDS	
PTCH2		WDR60	DisGeNet
KCNJ2		GJA1	DisGeNet
RAG1		OAT	
LMNA	DisGeNet	ZMPSTE24	
FAS	DisGeNet	TREX1	

In the prediction results of the above table, the candidate genes with known association were labeled in the **original DB**, and candidate genes marked with "*" indicate newly discovered associated genes, that is, there are not exist in dataset but records in the latest online database. The results show that our model has the ability to mine new disease gene associations, such as **OFD1-C057508** and **FLNA-C0678230**. Our model does not remember the existing associations in the original dataset, but predicts new candidate genes by mining the hidden patterns. This is very important, because it is difficult to mine new genes only by making a high score for the known associations. Therefore, our model can help to decipher the relationship between diseases and genes, which has certain biomedical significance

## Conclusions

In this paper, we use a new method to solve the early summation problem in heterogeneous network GNN model. By factoring the neighborhood subgraphs of homogeneous graphs transformed according to metapath, our proposed FactorHNE can mine a variety of semantic information in metapath complex patterns, and then generate excellent node embedding for link prediction through a double aggregation structure. The double aggregation structure first aggregates the semantic information in different factor graphs in a single metapath pattern, and then aggregates the semantic information in all metapath pattern by using the attention mechanism. In addition, we combine two loss functions in the optimization objective function of the model, and control the proportion of the two by weight coefficient to generate the most suitable node embedding for link prediction task. In the end, we compare the advantages and disadvantages of our model with a variety of baselines, and analyze some factors that affect the performance of the model by adjusting multiple parameters. Generally speaking, the FactorHNE model proposed by us shows good scalability and performance advantages.

## Materials and methods

### DataSet

We use the data set which is derived from [8] and contains four different node types: gene, disease, gene ontology (GO) and disease symptoms. They are shown in Table 3 and are available at https://github.com/xasdzxc/FactorHNE/tree/master/data.

**Table 3:** An overview of heterogeneous network dataset

Node	Number	Relation	Metapath
Gene (G)	21584	G–G|G–D|G–O	GG|GDG|GOG
Disease (D)	15030	D–G|D–S	DGD|DSD
GO (O)	14204	O–G	–
Symptom (S)	6540	S–D	–

We collected 130,820 disease-gene association from DisGeNet, 213,888 protein–protein interactions from Menche, 99,087 disease-symptom association from HPO and Orphanet, and 218,337 annotation records from STRING 10.

**Fig. 3:**
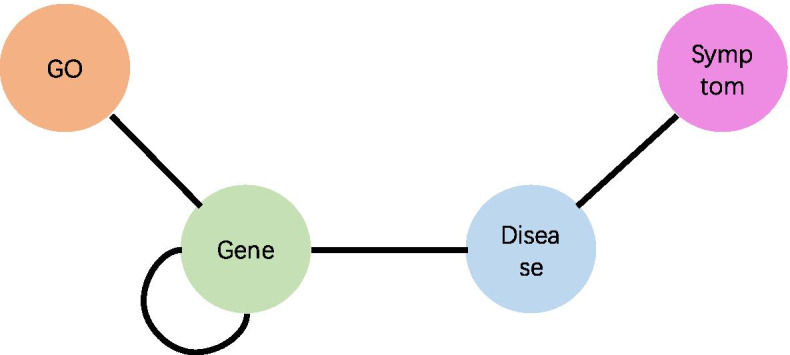
Metagraph for heterogeneous networks

All the initial values of edge weights in the heterogeneous network are set to 1. On this basis, the metagraph of the heterogeneous network we built is shown in Fig. 3:

### Model architecture

**Fig. 4:**
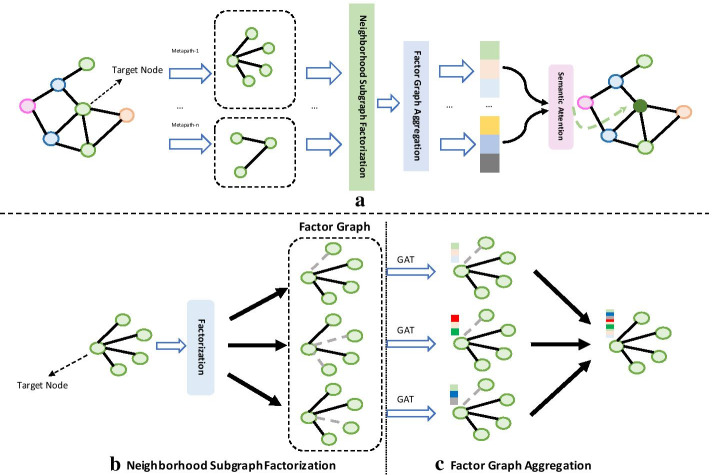
The overall architecture of FactorHNE. **a** Model global architecture; **b** neighborhood subgraph factor decomposition; **c** inter-metapath factor graph aggregation

In this section, we introduce the implementation principles and details of the individual components of the FactorHNE model. FactorHNE is composed of three main parts: neighborhood subgraph factorization, inter-metapath factor graph aggregation, and multi-metapath semantic aggregation. Figure 4 illustrates the overall framework of the FactorHNE model.

### Neighborhood subgraph factorization

For a heterogeneous graph $G = \left( {V,E} \right)$, It owns the node type set $A$ relational type set $R$, two mapping functions: $\varphi :{V} \to {A}$ and $\xi :E \to R$, and it has the property of $\left| A \right| + \left| R \right| > 2$. heterogeneous graph *G* contains a variety of types of nodes, different node contains its own features may not in the same space, such as, $d_{1}$ dimension node features and $d_{2}$ dimension another type node feature interact directly, even the dimension of the same case, is not reasonable, because of the feature space is different, just meaningless calculation. In order to solve this problem, we need to all types of nodes are projected onto the same vector space here. Our solution is for each node *v* of type *a* to design a linear transformation matrix $M_{a} \in {\mathbb{R}}^{{d^{\prime} \times d_{a} }}$, where $d^{\prime}$ denotes the dimension of all node type feature vector after the projection, and $d_{a}$ represents the original feature vector dimension of node type *a*, so we have the following procedure:

1 $$h_{v}^{{\prime}} = M_{a} \cdot x_{v}^{a}$$

where $x_{v}^{a}$ represents the original feature of node $v \in V_{a}$,$V_{a}$ represents the set of all nodes belonging to type $a \in A$, and $h_{v}^{{\prime}}$ represents the vector representation of node *v* after projected into the same space. In this way, it not only solves the problem caused by the heterogeneity of heterogeneous network, but also unifies the dimension of model input feature vector.

Next, we will define a collection of multiple metapath *M*, for one of the $m_{p} = a_{1} \to ^{{r_{1} }} a_{2} \to ^{{r_{2} }} \ldots a_{n - 1} \to ^{{r_{n - 1} }} a_{n}$ can be abbreviated to $a_{1} \cdot a_{2} \ldots a_{n - 1} \cdot a_{n}$, where the source node connects to the target node through a range of different nodes and relationships using a defined pattern, which called “metapath”, and for each $m_{l} \in M$, the heterogenous graph $G$ is converted into a homogeneous graph $g_{{m_{l} }}$, where $l$ is index of the different metapaths is included. For each homogeneous graph *g* from one metapath converts, before our neighbor information in aggregate phase to factorization so that can capture a variety of semantic information implied in metapath instance edge, the key idea is homogeneous graph based on the transformation of metapath only focus on two end node and a synthesis edge, which can cause early summarization issue. With the factorization step, the model can capture a variety of relations information implied in a single edge at a simple figure, so as to solve early summarization issue. For this issue, our solution is to reconstruct the edge weight of homogeneous graph *g* with the same operation for many times, based on the following formula:

2 $$W_{e} = \sigma \left( {S\left( {h_{v}^{{\prime}} ,h_{u}^{{\prime}} } \right)} \right)$$

where $W_{e}$ denotes new weight matrix after refactoring with *g* each edge, *e* is factor graph, $\sigma \left( \cdot \right)$ is sigmod function that used for standardize weights, $S\left( {h_{v}^{{\prime}} ,h_{u}^{{\prime}} } \right)$ compute a score between node *v* and *u* (we use a single layer MLP as the implementation) because the focus is on the edge, so maintaining the features of nodes, we can get the factor graph $G = \left( {W_{e} ,h^{{\prime }} } \right)$. If we just repeat this step to obtain multiple factor graphs, we will not be able to distinguish the information of each factor graph, which will only increase the stability of the model and not be able to mine the multiple semantic information contained in the single metapath edge. So we need to apply constraints that will include different information from each factor graph in order to obtain the rich semantic information in the metapath instance edge [17]. A discriminant loss function for any factor graph is added here, after any label information is included

3 $$Y_{e} = Softmax\left( {F\left( {EnCoder\left( {W_{e} ,h^{{\prime }} } \right)} \right)} \right)$$

For each factor graph, we first coded it according to its new edge weight matrix $W_{e}$ and the original node feature set *h*′ to obtain a form that is convenient for classifier $F\left( \cdot \right)$ processing. The classifier adopted is a single-layer full connection layer. Then, after standardization by the $Softmax$ layer, cross entropy is used to calculate the discriminant loss of multiple factor graphs

4 $${\mathcal{L}}_{Factor} = \frac{1}{N}\mathop \sum \limits_{i}^{N} \left( {\mathop \sum \limits_{y = 1}^{{N_{e} }} - {\mathbb{I}}\left( {e = y} \right){\text{log}}\left( {P_{i}^{e} \left[ y \right]} \right)} \right)$$

where $N$ represents the number of factor graphs, and $N_{e}$ represents the number of different labels contained in all factor graphs, which we set to $N_{e} = N$ in order to distinguish each factor graph. ${\mathbb{I}}\left( \cdot \right)$ represents the indicator function, and the probability that the $i$th factor graph with label $y$ represented by $P_{i}^{e} \left[ y \right]$. Through these operations above, several factor graphs containing different semantic information can be finally obtained, as shown in Fig. 4b.

### Inter-metapath factor graph aggregation

In order to include multiple semantic information from any of the factor graphs, we used neighbor information aggregation from each factor graph, and after combining the feature information from each of the factor graph to produce any of the specific node information from metapath, this part have two steps, shown in Fig. 4c.

As for the single factor graph *e*, we use the self-attention mechanism proposed in the work of Veličković et al. [15] to aggregate the neighbors of the target node on the factor graph *e*. Specifically, we first calculate the attention weight between the target node and neighbor $j \in N$, *N* represents the set of all neighbors, as shown in the formula follow

5 $$w_{ij}^{m} = Attention\left( {h_{i}^{{\prime}} ,h_{j}^{{\prime}} ,m} \right)$$

where $w_{ij}^{m}$ represents the attention weights from nodes $\left( {i,j} \right)$ in factor graph *e* with the metapath $m$ connection, and $Attention\left( \cdot \right)$ is used to integrate feature vectors from nodes $i$ and $j$ after projection with an attention vector, after the standardization of any neighbor attention weights from the target nodes from metapath $m$. the process is as follows

6 $$\alpha_{ij}^{m} = Softmax\left( {w_{ij}^{m} } \right) = \frac{{exp(w_{ij}^{m} )}}{{\mathop \sum \nolimits_{k = 1}^{\left| N \right|} {\text{exp}}(w_{ik}^{m} )}}$$

**Fig. 5:**
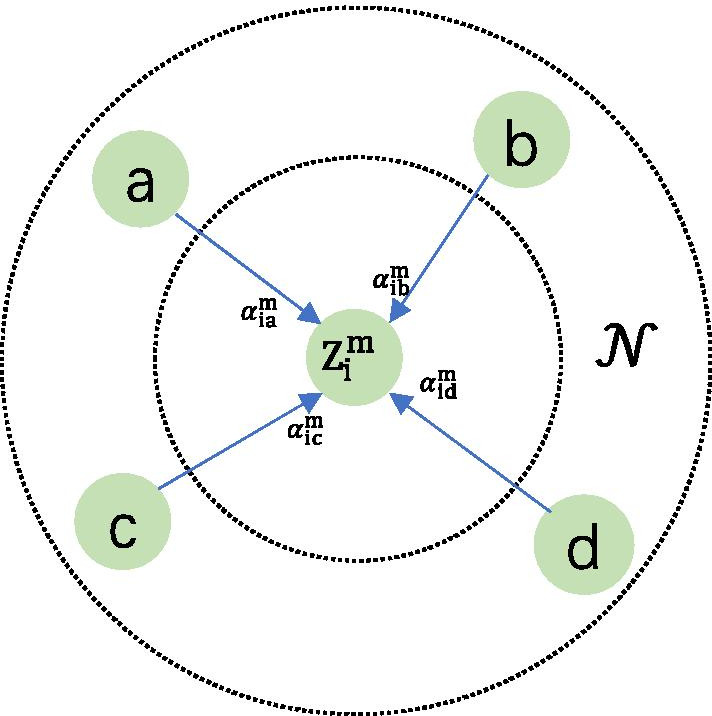
An example of node aggregation based on self-attention mechanism

Once the attention weights of all the neighbors have been generated, the aggregation operation can be performed, as shown in Fig. 5. The formula is as follows:

7 $$Z_{i}^{m} = \mathop \sum \limits_{j}^{\left| N \right|} \alpha_{ij}^{m} \cdot h_{j}^{{\prime}}$$

*N* here includes the target node itself. In order to guarantee the aggregation process can maintain stability, we adopted multi-head attention mechanism. Meanwhile, it still can expand capacity of the model is based on repeat $K$ times attention aggregation process, then $K$ results concatenate together in the end, all the factors graph generated feature vector concatenate together as a representation vector with metapath $m$

.

### Multi-metapath semantic aggregation

Previous sections illustrate a full process based on single metapath $m$, therefore, we need to integrate the semantic information and structural information from different metapath $\left\{ {m_{1} ,m_{2} , \ldots ,m_{l} } \right\}$. Now we have all node embedding set $\left\{ {Z_{v}^{{m_{1} }} ,Z_{v}^{{m_{2} }} , \ldots ,Z_{v}^{{m_{l} }} } \right\}$ generated by different metapath pattern. In particular, we averaged any of the target node embedding from any metapath as follows

8 $$P_{{m_{i} }} = \frac{1}{{\left| {V_{a} } \right|}}\mathop \sum \limits_{{v \in V_{a} }} {\text{tanh}}\left( {W_{a} Z_{v}^{{m_{i} }} + \varepsilon_{a} } \right)$$

where $W_{a}$ is a linear transformation matrix that specific to a certain node type *a*, $\varepsilon_{a}$ is corresponding to the bias of the linear transformation, both are trainable parameter. *V*_*a*_ denote all node of type *a* in homogeneous graph based on metapath $m_{i}$. Similar to the process of calculate weight neighbor's attention weight in section above, for each of the metapath $m_{i}$ attention while computing information fusion weights, the formula is as follows.

9 $$w_{{m_{i} }} = \left\langle {Q,P_{{m_{i} }} } \right\rangle$$

10 $$\omega_{{m_{i} }} = Softmax\left( {w_{{m_{i} }} } \right) = \frac{{{\text{exp}}\left( {w_{{m_{i} }} } \right)}}{{\mathop \sum \nolimits_{j = 1}^{l} {\text{exp}}\left( {w_{{m_{j} }} } \right)}}$$

11 $$H_{v} = \mathop \sum \limits_{{m_{i} \in M}} \omega_{{m_{i} }} \cdot Z_{v}^{{m_{i} }}$$

where $Q$ is an attention vector at the single metapath level, $\left\langle \cdot \right\rangle$ is an inner product, $\omega_{{m_{i} }}$ is an attention weight of metapath $m_{i}$ and *H*_*v*_ is an embedding vector of the final heterogeneous network node *v* with multi-metapath semantic information.

### Optimization

We aim to obtain a heterogeneous network embedding model that dedicated to disease-gene association prediction. Some previous models based on random walk usually divide generated node embedding and link prediction into two parts, which leads to final node embedding lack of optimization information of link prediction task. Our FactorHNE model benefits from the underlying architecture of neural network and can combine link prediction task in an end-to-end model. We calculate a similarity score by designing the decoder for the node pairs that need to be predicted. Here we directly set the decoder as the inner product, and then we have

12 $$Score_{gd} = \sigma \left( {\left\langle {H_{g} ,H_{d} } \right\rangle } \right)$$

For the loss function of the model, we adopt the binary cross entropy function, the specific form is shown as follows

13 $$L_{Pred} = - \mathop \sum \limits_{{\left( {g,d} \right) \in {\Phi }}} {\text{log}}\left( {Score_{gd} } \right)) - \mathop \sum \limits_{{\left( {g,d} \right) \in {\Phi }^{ - } }} {\text{log}}( - Score_{gd} )$$

where ${\Phi }$ is the edge set exist in original network dataset, ${\Phi }^{ - }$ is a set of gene and disease node pairs from negative sampling [21] in original dataset, so that our model can enjoy optimization based on downstream tasks. we mentioned before, set up a loss function $L_{Factor}$ for graph factorization and as a result, we disposed by setting the weight $\gamma$ to control the balance of the loss function from two parts, we have the final optimization goal as follows

14 $$Loss = L_{Pred} + \gamma \cdot L_{Factor}$$

## Data Availability

All the datasets are available at: https://github.com/xasdzxc/FactorHNE/tree/master/data. All additional files are available at: https://github.com/xasdzxc/FactorHNE.

## Abbreviations

ROC: Receiver operating characteristic
AUC: Area under the curve
P–R: Precision–recall
AP: Average precision
GNN: Graph neual network
